# Impact of chronic stress exposure on cognitive performance incorporating the active and healthy aging (AHA) concept within the cross-sectional Bern Cohort Study 2014 (BeCS-14)

**DOI:** 10.1007/s00404-021-06289-z

**Published:** 2021-11-06

**Authors:** Deborah Arifi, Norman Bitterlich, Michael von Wolff, Dagmar Poethig, Petra Stute

**Affiliations:** 1 Department of Obstetrics and Gynecology, Inselspital Bern, Bern, Switzerland; 2 Medizin & Service GmbH, Chemnitz, Germany; 3 European Association on Vitality and Active Aging eVAA E.V, Leipzig, Germany; 4 Gynecologic Endocrinology and Reproductive Medicine, University Women’s Hospital, Friedbühlstrasse 19, 3010 Bern, Switzerland

**Keywords:** Chronic stress, Cognition, European Innovation Partnership on Active and Healthy Ageing (EIP-AHA), Bio-functional status (BFS), Bio-functional age (BFA), Bern Cohort Study 2014 (BeCS-14)

## Abstract

**Purpose:**

This study aims to verify that the mental-cognitive domain of the validated generic bio-functional status (BFS)/bio-functional age (BFA) assessment tool, incorporating the concept of Active and Healthy Ageing (AHA), reflects cognitive performance. In addition, the effects of chronic stress exposure on the mental-cognitive BFS/BFA should be investigated.

**Methods:**

The study was carried out as a monocenter, cross-sectional, observational, non-interventional trial (Bern Cohort Study 2014, BeCS-14) with the participation of 147 non-pediatric, non-geriatric subjects. All participants followed a standardized battery of biopsychosocial assessments consisting of BFS/BFA, a validated cognitive performance test battery (Inventar zur Gedächtnisdiagnostik; IGD) and a validated questionnaire for the assessment of chronic stress (Trier Inventory for the assessment of Chronic Stress; TICS), respectively.

**Results:**

Mean cognitive performance was average and higher in younger or better educated individuals. The BFA of the participants was 7.8 ± 7.8 year-equivalents below their chronological age. The mental-cognitive BFS/BFA assessment correlated well with the validated questionnaire for cognition assessment, the IGD. Further, three TICS subdomains (work overload (*r* =  − 0.246, *p* = 0.003), work discontent (*r* =  − 0.299, *p* = 0.006) and pressure to succeed (*r* =  − 0.274, *p* < 0.001)), reflecting mainly work-related stress, showed a significant negative correlation with the mental-cognitive BFS/BFA.

**Conclusions:**

Our study shows that the BFS/BFA assessment tool follows European Innovation Partnership on Active and Healthy Ageing (EIP-AHA) requirements. Further, we could demonstrate that higher levels of chronic work-related stress may be associated with poorer mental-cognitive performance and a pro-aging state indicating that cognitive impairments can be reduced by stress management interventions.

**Supplementary Information:**

The online version contains supplementary material available at 10.1007/s00404-021-06289-z.

## Introduction

The European Innovation Partnership on Active and Healthy Ageing (EIP on AHA), launched by the European Commission in 2012, addresses the challenge of demographic change in Europe aiming at better prospects for the aging population in terms of quality of life, overall health and well-being [1, 2]. Several items such as functioning (individual capability and underlying body systems), wellbeing, activities and participation, and diseases including chronic non-communicable diseases are part of the corresponding AHA framework [3].

Based on two cross-sectional studies, the Leipzig Cohort Study 1984 (LeCS-84) [4] and the Bern Cohort Study 2014 (BeCS-14) [5], we have developed our complex generic AHA assessment tool. This validated, non-invasive tool contains physical, mental-cognitive, emotional and social domains and allows for calculating the so-called bio-functional status (BFS) and bio-functional age (BFA), respectively. It follows EIP-AHA requirements and incorporates the International Classification of Functioning, Disability and Health of the WHO.

In our previous BeCS-14 analyses we found that, first, the BFS cognitive-mental function subdomain correlated well with the validated questionnaire for cognition assessment, IGD [6]. Secondly, we found the BFS/BFA assessment tool to reflect chronic stress exposure with higher chronic stress exposure being associated with bio-functional pro-aging in both sexes [7].

The present BeCS-14 analysis aimed to (1) verify the correlation between the BFS cognitive-mental function subdomain and the IGD by expanding the study population, (2) investigate the impact of age on the correlation between the BFS cognitive-mental function subdomains and the IGD, and (3) investigate the impact of chronic stress exposure and its subdomains on the BFS cognitive-mental function.

## Materials and methods

### Study population

Between 04.03.2012 and 04.07.2014 a group of 147 German-speaking women and men aged 18–70 years were recruited at the Department of Obstetrics and Gynecology, Inselspital Bern, Switzerland. Recruitment was performed by the principal investigator, the study nurse and 14 doctoral students of the medical school, University Bern, via personal contact (patients, colleagues, family, friends) and online advertisement (Internet, intranet Inselspital Bern, social media). The exclusion criteria included pregnancy, acute diseases (e.g., fever, acute pain syndrome), and illiteracy. The study protocol was approved by the Cantonal Ethics Committee Bern (Ref.-Nr. KEK-BE: 023112). Written informed consent was obtained from each participant.

### Study design

This was a monocenter, cross-sectional, observational, non-interventional trial [5]. All participants within BeCS-14 followed a standardized battery of assessments consisting of a personal and family history, bio-functional status (BFS) and bio-functional age (BFA), and validated questionnaires for depression and anxiety (HADS) [8], health-related quality of life (SF-36) [9] and chronic stress exposure (TICS) [10], respectively. Participants were further divided into four additional assessment subgroups addressing “nutrition” by AD-EVA [11] and PATEF [12] (subgroup 1), “employees’ health” by IMPULS [13] (subgroup 2), “stress” by heart rate variability [14] (subgroup 3) and “cognition” by the validated test battery IGD [15] (subgroup 4).

### Assessment procedures

#### Personal and family history

The assessment of personal and family history included age, social status, lifestyle, and job status. Further, it comprised information about malignancy, cardiovascular disease, breathing disorder, abdominal and urogenital disease, metabolic disorder, skin and/or hair disease, neuromuscular and psychiatric disorder as well as bone and joint disease.

#### Bio-functional status (BFS), bio-functional age (BFA) and mental-cognitive BFS subdomain

The BFS was assessed by a comprehensive test battery developed by Poethig et al. and reported by others [1, 5, 15–17], respectively. It is a validated age- and sex-specific tool (objectivity 0.96, reliability 0.93, female age validity: total age correlation 85.2%; total age communality in the main factor 76.3%). The BFA is based on a sex-specific regression and factor analysis of functional age [4, 16–18]. Table 1 represents the single items (M1–M12) covering the BFS cognitive-mental function subdomain. Detailed descriptions of the individual BFS subdomains can be found in our pre-study [6] (Table 1).

**Table 1:** Mental-cognitive subdomain of the bio-fucntional status (BFS) and Inventar zur Gedächtnisdiagnostik (IGD) test battery

BFS subtests (M1–M12) and subdomains (constructed out of subtests M1–M12)	IGD subtests (A1–A12) and subdomains (constructed out of subtests A1–A12)
Optical reaction time (M1): optical response (10 times), average time [ms]	Prospective memory (A1): this subtest stands out from other cognitive batteries. The task is to save two different tasks which have to be fulfilled without being recalled on. Maximal 24 points [forward, explicit, verbal memory]
Pursuing reaction time (M2): pursuing reaction time (10 times), average time [ms]	Forward digit span (A2): short-term memory on forward digit spans with increasingly longer series. Maximal 24 points [short-term, explicit, verbal memory]
Acoustic reaction time (M3): acoustic response (10 times), average time [ms]	Verbal working memory (A3): words with a specific attribute have to be selected and retained (consciously and unconsciously) out of a wordlist. Maximal 21 points. [short-term, explicit, verbal memory]
Verbal reaction time (M4): [s]	Visual working memory (A4): Objects in different position and orientation have to be memorized. Maximal 21 points. [Short-term, explicit, visuospatial memory]
Cognitive reaction time (M5): [s]	Executive function (A5): shift of attention between two different patterns that have to be retained and afterwards transformed verbally. Maximal 27 points [short-term, explicit, visual-verbal memory]
Cognitive switching capability (M6): [s]	Verbal learning (A6): recognition of words out of semantic similar words in the original text. Maximal 20 points [intermediate, explicit, verbal memory]
Ability to concentrate, time (M7): [s]	Visual learning (A7): Recognition of figures and completing the missing parts. Maximal 20 points. [intermediate, explicit, visual memory]
Ability to concentrate, mistakes (M8): number of mistakes [*n*]	Pair association (A8): learning shape/color pairs. Maximal 21 points [intermediate, explicit, visual–verbal memory]
Strategic thinking (M9): total time [s]	Delayed recognition: wordlist (A9): recognition of consciously and unconsciously learned words from subtest A3. Maximal 17 points [longer-term, explicit, verbal memory]
Memory performance (M10): number of repetition mistakes [*n*]	Delayed recognition: text (A10): content reproduction from the text read in subtest A6 through answering questions. Maximal 20 points [longer-term, explicit, verbal memory]
Orientation capability (M11): number of mistakes [*n*]	Delayed recognition: figures (A11): figure recognition from subtest A7. Maximal 20 points [longer- term, explicit, visual memory]
Changeover capability (M12): average time for one step [s]	Priming (A12): completing words that have been memorized unconsciously. Maximal 24 points [intermediate, implicit, verbal memory]
Color-word test by Stroop, modified: M4–M6	Short-term memory/working memory (ST/WM): A2–A5. Maximal 72 points
Concentration–time test (Landolt): M7, M8	Learning (L): A6–A8. Maximal 61 points
Stepping-stone-maze test (Poegelt und Roth): M9–M12	Verbal memory (VeM): A3, A6, A9, A10. Maximal 78 points
	Visual memory (ViM): A4, A7, A11. Maximal 61 points
	Delayed recall (DR): A9, A10, A11. Maximal 57 points
	Total memory (Total): A1–A12. Maximal 238 points

#### Assessment of cognitive performance

Cognitive performance was examined by section A of the German validated test battery IGD (Inventar zur Gedächtnisdiagnostik) [15]. It includes 12 subtests (A1–A12) which can be assigned to six cognitive subdomains: short-term memory/working memory (ST/WM), learning (L), verbal memory (VeM), visual memory (ViM), delayed recall (DR) and total memory (T) (Table 1). The IGD has been normed for age (also represented as percentile ranks), while sex and level of education were not considered to be significant and were thus not taken into account [15].

#### Assessment of chronic stress exposure

Chronic stress exposure was assessed by TICS (Trierer Inventar zum chronischen Stress), a standardized, validated questionnaire (Cronbach alpha 0.9) [10]. Nine aspects of chronic stress are distinguished; work overload, social overload, pressure to perform, work discontent, excessive demands at work, lack of social recognition, social tensions, social isolation and chronic worrying. In addition, a global value for chronic stress exposure within the past 3 months is represented by a standardized screening scale for chronic stress (SSCS), covering five out of the nine chronic stress domains. The total score ranges from 0 to 48 points providing three subcategories of perceived chronic stress intensity: below average stress (0–11 points), above average stress (12–22 points) and extreme stress (> 22 points) [19].

### Statistical methods

Statistical analysis was performed with SPSS (Statistical Package for Social Sciences) version 25.0. The descriptive statistics contained the calculation of the mean, standard deviation and for ordinal parameters the percentages. Statistical comparison of population subgroups were performed with the non-parametric Fisher, Kruskal–Wallis- and Mann–Whitney *U* test. For correlation analysis, a two-sided Pearson product–moment correlation coefficient test was used. A *p* value < 0.5 was considered statistically significant. In contrast to the IGD and the TICS, the BFS does not provide percentile ranks. For this reason, we used the raw and not age-adjusted scores for statistical comparison between IGD and BFS and between TICS and BFS, respectively.

## Results

### Characteristics of the cohort

#### Age, lifestyle, social and job status

Overall, 624 subjects were included in the BeCS-14. Of those, 147 subjects (37.4% male, 62.6% female) took part in subgroup “cognition” (supplementary table 1). Mean age was 38.0 ± 15.3 years. About one-third (31.3%) had a degree from university or advanced technical college, respectively. Most participants reported being employees (54.5%), 16.1% being in leading positions and 22.4% being students. The monthly gross income was less than 5000 CHF for 50.0%. The majority (80.1%) of the participants reported a regular alcohol consumption at least twice a month, 35.9% at least twice a week, and 23.1% had at least one drink per day. The majority were never-smoker (63.9%), physically active (till sweating) at least once a week (75.5%) and most participants reported sleeping between 7 and 8 h a night (72.1%). When comparing age-based subpopulations, there were statistically significant differences within the categories education, job status, monthly gross income and physical activity. Age group 2 (26–49 years) showed the highest percentage of participants with a degree from the university or advanced technical college. In general, job status and monthly gross income were higher in participants aged ≥ 26 years, whereas physical activity was significantly lower in participants aged ≥ 50 years.

#### Personal history

Life-threatening events were reported by two participants (1.4%; stroke *n* = 1, myocardial infarction *n* = 1). The prevalence of cardiovascular risk factors was 7.2% for hypertension, 8.6% for dyslipidemia, and 0.7% for diabetes mellitus. Five participants (3.7%) suffered from a malignant disease, 14.2% from depression. The prevalence of musculoskeletal disease was 13.6% (muscular disease *n* = 1, rheumatoid arthritis *n* = 1, arthrosis *n* = 12, osteoporosis *n* = 5). Comparison of age-based subpopulations showed statistically significant differences for sleep apnea, dyslipidemia, malignant disease, arthrosis and osteoporosis with a higher prevalence in subjects aged ≥ 50 years.

### Bio-functional status (BFS) and bio-functional age (BFA)

Table 2 presents the results for individual BFS items. Of those above age 35 years, the mean difference between the chronological and bio-functional age (Age-BFA-index) was 7.8 ± 7.8 years, indicating that the bio-functional age of the participants was about 8 year-equivalents below their chronological age (Table 2).

**Table 2:** Cognitive performance assessed by BFS (M1–M12)

Cognitive and mental function (BFS)	Total (*n* = 147) Mean (± SD)	≤ 25 (*n* = 45) Mean (± SD)	26–49 (*n* = 56) Mean (± SD)	≥ 50 (*n* = 44) Mean (± SD)
M1^1^	237.87 (36.71) ms	272.79 (23.21) ms	264.42 (31.27) ms	285.91 (48.67) ms
M2^2^	64.35 (29.92) ms	52.62 (20.24) ms	56.61 (23.34) ms	85.09 (34.45) ms
M3^3^	274.00 (40.72) ms	283.67 (22.46) ms	276.74 (47.08) ms	261.51 (42.90) ms
M4^4^	11.14 (1.96) s	10.52 (1.17) s	10.48 (1.43) s	12.55 (2.38) s
M5^5^	13.20 (2.32) s	12.54 (1.48) s	12.42 (1.75) s	14.80 (2.77) s
M6^6^	23.98 (10.74) s	19.60 (3.94) s	20.81 (3.77) s	32.09 (15.62) s
M7^7^	127.90 (41.85) s	111.58 (27.23) s	119.83 (39.09) s	153.35 (45.35) s
M8^8^	1.28 (1.73)	0.71 (1.24)	1.33 (1.78)	1.73 (1.95)
M9^9^	143.42 (62.94)	120.89 (24.56)	130.81 (50.37)	180.44 (83.62)
M10^10^	94.78 (21.39)	90.36 (6.92)	96.25 (28.80)	97.04 (18.96)
M11^11^	48.85 (13.08)	45.19 (5.76)	48.49 (16.10)	52.71 (12.93)
M12^12^	1.10 (0.36)	0.90 (0.20)	0.90 (0.21)	1.20 (0.52)

1: optical reaction time, 2: pursuing reaction time, 3: acoustic reaction time, 4: verbal reaction time, 5: cognitive reaction time, 6: cognitive switching capability, 7: ability to concentrate (time), 8: ability to concentrate (mistakes), 9: strategic thinking, 10: memory performance, 11: orientation capability, 12: changeover capability

### Cognitive performance

Cognitive performance was assessed by the BFS cognitive-mental function subdomain (M1–M12) (Table 2) and the IGD (subtests A1-A12, subdomains total, ST/WM, L, VeM, ViM, DR) (supplementary table 3), respectively. According to the IGD, the age-adjusted mean performance percentile rank was 52.9 ± 27.7 (range 0–100). The results of the BFS cognitive-mental function subdomain and the IGD subdomains were then adjusted for age, sex and education (supplementary tables 2, 4).

When adjusting the BFS cognitive-mental function subdomain for age, there were significant differences between age groups for all subdomains. In general, age group 3 (≥ 50 years) performed significantly worse than age group 1 (≤ 25 years) (M2, M4 -M12) and age group 2 (26–49 years) (M1, M2, M4, M5, M6, M7, M9, M11, M12). In contrast, differences between age group 1 (≤ 25 years) and 2 (26–49 years) were mostly non-significant (M2–M12). Similarly, the IGD showed significant differences between age group 3 (≥ 50 years) and age group 2 (26–49 years) resp. age group 1 (≤ 25 years) in subtests A1–A7, A9–A12 and all subdomains (Total, ST/WM, L, VeM, ViM, DR), while differences between age group 1 (≤ 25 years) and 2 (26–49 years) were mostly non-significant.

When adjusting the BFS cognitive-mental function subdomain for education level, there were significant group differences for verbal reaction time (M4), cognitive reaction time (M5), cognitive switching capability (M6), ability to concentrate (M8) and strategic thinking (M9), respectively. In general, subjects with the highest education level (university/federal institute of technology (ETH)/college of higher education) performed best, while subjects with the lowest education level (secondary school/district school) showed the worst results. In the IGD a similar pattern was observed. For all but one subdomain (prospective memory (A1)), educational level had a significant impact on IGD subdomains.

When adjusting the BFS cognitive-mental function subdomain for sex, there were almost no differences. Only for pursuing reaction time (M2), women performed significantly better than men. Similarly, sex only had a minor impact on IGD subdomains.

### Correlation analysis between the BFS cognitive-mental function subdomain and a validated cognitive performance test battery (IGD)

To prove the presentation of cognitive performance by the cognitive-mental BFS subdomain, a correlation analysis between the BFS and the validated IGD was performed, analogous to our preliminary study but with an expansion of the study population (Table 3). We found a significant inverse correlation between optical reaction time (M1), optical pursuing reaction time (M2), verbal reaction time (M4), cognitive reaction time (M5), cognitive switching capability (M6), strategic thinking (M9), changeover capability (M12), Stroop Test (M4–M6) and Stepping-stone-maze test (M9–M12) with total IGD and all IGD subdomains. This suggests that a person with better total memory (Total), short-term and working memory (ST/WM), learning ability (L), verbal memory (VeM), visual memory (ViM) and delayed recall (DR) performed better in these BFS cognitive-mental function subdomains. For the ability to concentrate (M7, M8), respectively, the Landolt test (M7–M8), memory performance (M10) and orientation capability (M11), all results of the total study population, correlated inversely with the IGD subdomains; however, some of these were non-significant; participants with a higher ability to concentrate (M7, M8) showed significantly better total memory (T), short-term and working memory (ST/WM), learning capacity (L) and visual memory (ViM), whereas for verbal memory (VeM) and delayed recall (DR), the correlation was inverse but not significant. In contrast, acoustic reaction time (M3) did not or positively correlated with IGD subdomains.

**Table 3:** Correlation analysis between the mental-cognitive BFS and a validated cognitive performance test battery (IGD)

IGD subdomains	Age (years)	Correlation coefficient (*r*) *p* value (*p*)	Cognitive and mental function (BFS)														
			M1^1^	M2^2^	M3^3^	M4^4^	M5^5^	M6^6^	M7^7^	M8^8^	M9^9^	M10^10^	M11^11^	M12^12^	Stroop test (M4–M6)	Landolt test (M7–M8)	Stepping-stone-maze test (M9–M12)
Total^13^	Total	*r*	− 0.442**	− 0.440**	− 0.940	− 0.418**	− 0 − 516**	− 0.594**	− 0.197*	− 0.191*	− 0.524**	− 0.041	− 0.090	− 0.570**	− 0.617**	− 0.205*	− 0.415**
		*p*	< 0.001	< 0.001	0.261	< 0.001	< 0.001	< 0.001	0.018	0.022	< 0.001	0.624	0.281	< 0.001	< 0.001	0.014	< 0.001
ST/WM^14^	Total	*r*	− 0.231**	− 0.430**	0.213*	− 0.451**	− 0.494**	− 0.576**	− 0.278**	− 0.177*	− 0.484**	− 0.187*	− 0.202*	− 0.438**	− 0.604**	− 0.287**	− 0.438**
		*p*	0.005	< 0.001	0.010	< 0.001	< 0.001	< 0.001	0.001	0.034	< 0.001	0.025	0.015	< 0.001	< 0.001	< 0.001	< 0.001
L^15^	Total	*r*	− 0.205*	− 0.474**	0.272**	− 0.351*	− 0.431*	− 0.499**	− 0.249**	− 0.173*	− 0.488**	− 0.204*	− 0.243**	− 0.425**	− 0.518**	− 0.257**	− 0.452**
		*p*	0.014	< 0.001	0.001	< 0.001	< 0.001	< 0.001	0.003	0.038	< 0.001	0.014	0.003	< 0.001	< 0.001	0.002	< 0.001
VeM^16^	Total	*r*	− 0.188*	− 0.317**	0.098	− 0.306**	− 0.296**	− 0.359**	− 0.135	− 0.036	− 0.343**	− 0.159	− 0.194*	− 0.284**	− 0.378**	− 0.137	− 0.325**
		*p*	0.024	< 0.001	0.241	< 0.001	< 0.001	< 0.001	0.106	0.667	< 0.001	0.057	0.020	0.001	< 0.001	0.101	< 0.001
ViM^17^	Total	*r*	− 0.246**	− 0.482**	0.302*	− 0.347**	− 0.463**	− 0.572**	− 0.218**	− 0.256**	− 0.488**	− 0.188*	− 0.243**	− 0.425**	− 0.581**	− 0.230**	− 0.448**
		*p*	0.003	< 0.001	< 0.001	< 0.001	< 0.001	< 0.001	0.009	0.002	< 0.001	0.024	0.003	< 0.001	< 0.001	0.006	< 0.001
DR^18^	Total	*r*	− 0.230**	− 0.395**	0.149	− 0.287**	− 0.333**	− 0.425**	− 0.075	− 0.116	− 0.331**	− 0.024	− 0.145	− 0.322**	− 0.434**	− 0.080	− 0.275**
		*p*	0.006	< 0.001	0.074	< 0.001	< 0.001	< 0.001	0.374	0.116	< 0.001	0.773	0.083	< 0.001	< 0.001	0.343	0.001

1: optical reaction time, 2: pursuing reaction time, 3: acoustical reaction time, 4: verbal reaction time, 5: cognitive reaction time, 6: cognitive switching capability, 7: ability to concentrate (time), 8: ability to concentrate (mistakes), 9: strategic thinking, 10: memory performance, 11: orientation capability, 12: changeover capability, 13: total memory (A1–A12), 14: short-term memory/working memory (A2–A5), 15: learning (A6–A8), 16: verbal memory (A3, A6, A9, A10), 17: visual memory (A4, A7, A11), 18: delayed recall (A9–A11)

**The correlation is significant at the level 0.01 (2-sided)

*The correlation is significant at the level 0.05 (2-sided)

In a second step, correlation analysis between the BFS cognitive-mental function subdomain and IGD was performed for three age subgroups (≤ 25 years, 26–49 years, ≥ 50 years) (supplementary table 5). For subjects aged ≤ 25 years, the correlation between the BFS cognitive-mental function subdomain and IGD was weakest with five BFS subdomain items being significantly correlated to IGD subdomains. In detail, acoustic reaction time (M3), cognitive switching capability (M6), strategic thinking (M9), orientation capability (M11) and the Stroop test (M4–M6) were significantly correlated to most IGD subdomains (Total, ST/WM, L, VeM, ViM).

For subjects aged 26–49 years, the correlation between the BFS cognitive-mental function subdomain and IGD was stronger with seven BFS subdomain items showing significant correlations: optical pursuing reaction time (M2), acoustic reaction time (M3), cognitive switching capability (M6), strategic thinking (M9), memory performance (M10), Stroop test (M4–M6) and Stepping-stone-maze test (M9–M12). The strongest correlation was found for subjects aged ≥ 50 years. Here, ten BFS cognitive-mental function subdomain items showed significant correlations to IGD subdomains, with optical reaction time (M1), optical pursuing reaction time (M2), strategic thinking (M9) and changeover capability (M12) being significantly correlated to all IGD subdomains. Similarly, cognitive switching capability (M6), Stroop test (M4–M6) and Stepping stone-maze test (M9–M12) were significantly correlated to all IGD subdomains except for VeM. Acoustic, verbal and cognitive reaction time (M3, M4, M5) were significantly correlated to at least one IGD subdomain (Table 3).

### Chronic stress exposure

Table 4 presents the results for chronic stress exposure assessed by TICS. The chronic stress level overall (SSCS) of the BeCS-14 subgroup “cognition” was comparable to the mean values (T50) of the TICS reference population, as well as the stress levels in the subdomains work overload, work discontent, lack of social recognition, social isolation and social tensions. However, chronic stress exposure by chronic worrying and pressure to perform were lower, whereas excessive demands at work and social overload were higher in the BeCS-14 subgroup “cognition”.

**Table 4:** Chronic stress assessed by Trierer Inventar zum chronischen Stress (TICS)

TICS subdomain	Total Mean (± SD)	≤ 25 (*n* = 45) Mean (± SD)	26–49 (*n* = 56) Mean (± SD)	≥ 50 (*n* = 44) Mean (± SD)	T50 (reference cohort)
Chronic worrying	4.92 (2.86)	5.16 (3.06)	4.90 (3.16)	4.70 (2.29)	14
Excessive demands at work	5.71 (3.33)	6.02 (3.00)	5.88 (3.75)	5.19 (3.06)	4.5
Work overload	11.85 (5.80)	11.40 (5.20)	13.26 (5.56)	10.45 (6.33)	12.5
Work discontent	9.46 (4.73)	11.00 (4.80)	10.02 (4.94)	7.20 (3.45)	9
Pressure to perform	15.75 (5.67)	14.43 (4.13)	18.12 (5.99)	13.79 (5.44)	17
Lack of social recognition	4.12 (2.73)	4.09 (2.28)	4.61 (3.12)	3.45 (2.49)	4
Social overload	9.17 (4.24)	7.47 (4.67)	10.02 (4.15)	9.70 (3.48)	7
Social isolation	5.68 (3.63)	6.11 (3.82)	5.93 (3.71)	4.91 (3.26)	5
Social tensions	5.08 (3.18)	4.81 (3.38)	5.54 (3.21)	4.73 (2.93)	5
Screening Scale (SSCS)	13.98 (6.61)	14.16 (6.70)	14.72 (6.93)	12.84 (6.07)	13

The TICS’ results (SSCS, subdomains) were then adjusted for age, sex and education (supplementary table 6). With respect to age, there were significant differences for chronic stress exposure overall (TICS-SSCS) and for the subdomains work overload, work discontent, pressure to perform, lack of social recognition and social overload. In general, chronic stress exposure was highest in age group 2 (26–49 years). Compared to their older counterparts (≥ 50 years), they had a significantly higher chronic stress level in the TICS subdomains work overload, work discontent, pressure to perform and lack of social recognition. Compared to their younger counterparts (≤ 25 years), chronic stress level was significantly higher in the TICS subdomain pressure to perform. On the contrary, chronic stress exposure was highest for the youngest age group (≤ 25 years) in the TICS subdomain work overload. Interestingly, sex did not have an impact on chronic stress exposure (SSCS, subdomains). For education, we found significant differences for chronic stress exposure overall (TICS-SSCS) and the subdomains excessive demands at work, work discontent, pressure to perform and social isolation with higher educated participants (Matura or university-degree/advanced technical college degree) showing higher chronic stress levels (Table 4).

### Correlation analysis between the BFS cognitive-mental function subdomain and chronic stress exposure (TICS)

A correlation analysis between the BFS cognitive-mental function subdomain and TICS was performed to assess the impact of chronic stress exposure on cognitive function (Table 5). None but one (acoustic reaction time, M3) of the BFS cognitive-mental function subdomain items showed a significant correlation to overall chronic stress exposure (SSCS-TICS). When differentiating for TICS subdomains, this was also true for the subdomains lack of social recognition, social overload, social isolation and social tensions, respectively.

**Table 5:** Correlation analysis between the mental-cognitive BFS domain and chronic stress exposure (TICS)

TICS subdomains	Correlation coefficient (*r*) *p* value (*p*)	Cognitive and mental function (BFS)														
		M1^1^	M2^2^	M3^3^	M4^4^	M5^5^	M6^6^	M7^7^	M8^8^	M9^9^	M10^10^	M11^11^	M12^12^	Stroop test (M4-M6)	Landolt test (M7-M8)	Stepping-stone-maze test (M9-M12)
Chronic worrying	*r*	0.203*	0.069	0.184*	− 0.038	0.021	− 0.012	− 0.079	0.144	0.006	− 0.033	0.015	0.018	− 0.012	− 0.073	− 0.001
	*p*	0.015	0.410	0.028	0.655	0.801	0.882	0.350	0.086	0.939	0.692	0.863	0.833	0.890	0.386	0.988
Excessive demands at work	*r*	0.152	− 0.021	0.213*	− 0.090	− 0.015	− 0.083	− 0.035	0.104	− 0.078	− 0.33	0.018	− 0.076	− 0.081	− 0.030	− 0.064
	*p*	0.074	0.803	0.012	0.289	0.856	0.332	0.686	0.223	0.359	0.702	0.837	0.373	0.342	0.722	0.456
Work overload	*r*	− 0.082	− 0.150	0.170*	− 0.176*	− 0.211*	− 0.233**	− 0.168*	0.093	− 0.127	0.011	0.015	− 0.149	− 0.246**	− 0.165	− 0.090
	*p*	0.336	0.077	0.045	0.037	0.012	0.006	0.047	0.274	0.135	0.897	0.858	0.078	0.003	0.052	0.292
Work discontent	*r*	0.064	− 0.177*	0.174*	− 0.188*	− 0.127	− 0.175*	− 0.141	0.054	− 0.299**	− 0.125	− 0.132	− 0.186*	− 0.187*	− 0.139	− 0.222**
	*p*	0.450	0.034	0.038	0.024	0.130	0.036	0.094	0.520	0.006	0.136	0.115	0.026	0.025	0.098	0.008
Pressure to perform	*r*	− 0.090	− 0.200*	0.086	− 0.240**	− 0.203*	− 0.274**	− 0.157	0.059	− 0.280**	− 0.064	− 0.064	− 0.076	− 0.286**	− 0.155	− 0.237**
	*p*	0.289	0.018	0.310	0.004	0.016	0.001	0.063	0.488	0.001	0.449	0.449	0.368	0.001	0.066	0.005
Lack of social recognition	*r*	0.026	− 0.120	0.113	− 0.058	− 0.136	− 0.108	− 0.053	0.011	− 0.080	− 0.026	− 0.039	− 0.039	− 0.117	− 0.053	− 0.072
	*p*	0.760	0.154	0.178	0.492	0.105	0.200	0.528	0.895	0.340	0.758	0.758	0.643	0.165	0.530	0.391
Social overload	*r*	0.072	− 0.062	0.007	− 0.016	− 0.038	− 0.045	− 0.002	0.1072	− 0.08	0.022	0.080	− 0.127	− 0.044	0.002	− 0.043
	*p*	0.394	0.462	0.930	0.852	0.655	0.594	0.979	0.204	0.331	0.794	0.343	0.130	0.600	0.979	0.607
Social isolation	*r*	0.093	− 0.060	0.139	− 0.093	− 0.077	− 0.100	− 0.073	− 0.035	− 0.057	− 0.074	− 0.032	− 0.044	− 0.105	− 0.075	− 0.066
	*p*	0.272	0.475	0.098	0.270	0.358	0.236	0.383	0.676	0.495	0.379	0.704	0.598	0.211	0.372	0.432
Social tensions	*r*	0.132	− 0.018	0.154	− 0.002	0.049	0.034	0.015	0.129	− 0.021	− 0.001	0.014	− 0.020	0.035	0.020	− 0.014
	*p*	0.115	0.835	0.067	0.984	0.561	0.691	0.863	0.124	0.807	0.988	0.872	0.814	0.682	0.813	0.872
Screening Scale for Chronic Stress (SSCS)	*r*	0.143	− 0.014	0.211*	− 0.109	− 0.084	− 0.105	− 0.098	0.138	− 0.082	− 0.042	− 0.003	− 0.077	− 0.113	− 0.093	− 0.072
	*p*	0.092	0.869	0.012	0.201	0.322	0.217	0.247	0.104	0.333	0.619	0.974	0.366	0.184	0.274	0.395

1: optical reaction time, 2: pursuing reaction time, 3: acoustic reaction time, 4: verbal reaction time, 5: cognitive reaction time, 6: cognitive switching capability, 7: ability to concentrate (time), 8: ability to concentrate (mistakes), 9: strategic thinking, 10: memory performance, 11: orientation capability, 12: changeover capability

**The correlation is significant at the level 0.01 (2-sided)

*The correlation is significant at the level 0.05 (2-sided)

However, three TICS subdomains (work overload, work discontent, pressure to succeed) showed significant negative correlations to some BFS cognitive-mental function subdomains. In detail, work overload significantly negatively correlated with verbal and cognitive reaction time (M4, M5), cognitive switching capability (M6), ability to concentrate (M7) and the Stroop test (M4–M6), respectively. Work discontent showed the strongest negative correlation to the BFS with pursuing reaction time (M2), verbal reaction time (M4), cognitive switching capability (M6), strategic thinking (M9), changeover capability (12), Stroop test (M4–M6) and Stepping-stone-maze test (M9–M12) being significantly negatively correlated. Pressure to succeed showed a significant negative correlation to pursuing reaction time (M2), verbal and cognitive reaction time (M4, M5), cognitive switching capability (M6), strategic thinking (M9) as well as Stroop test (M4–M6) and Stepping-stone-maze test (M9–M12). This indicates that participants with higher chronic stress level, especially in the subcategories work overload, work discontent and pressure to succeed, i.e., predominantly work-related stress subdomains, showed a significantly negative correlation with the BFS cognitive-mental function subdomain and thus worse results than their less stressed counterparts.

Surprisingly, the BFS subdomains’ optical response (M1) and acoustic reaction time (M3) showed a significantly positive correlation with the TICS subdomains chronic worrying and excessive demands at work, work overload and work discontent, respectively. A higher chronic stress level in these subdomains is thus associated with a faster visual and optical reaction time.

TICS-SSCS and the difference between chronological and bio-functional age (Age–BFA index) were negatively (but not significantly) correlated (Pearson correlation − 0.12; *p* = 0.176). This was also true for the correlation between TICS subdomains and the age–BFA index. This finding indicates that higher chronic stress exposure was associated with bio-functional pro-aging in both sexes and thus underlines the results of our previous investigations [7] (Table 5).

## Discussion

In the current study, we were able to demonstrate that (1) mean cognitive performance level was average and higher in younger or better educated individuals, respectively; (2) mean difference between chronological age and bio-functional age (age–BFA index) in the BeCS-14 subgroup “cognition” was 7.8 ± 7.8 year equivalents; (3) the BFS cognitive-mental function subdomain (with expansion of the study population) reflected cognitive performance and thus corresponds to previous analysis [6]; (4) whereas correlation between BFA and IGD was found to be stronger for individuals aged ≥ 25 years; (5) chronic stress exposure in the BeCS-14 subgroup “cognition” was comparable to that of the TICS reference cohort; and (6) a higher chronic stress exposure was associated with bio-functional pro-aging, although the correlation was found to be not significant (corresponding to our previous study [7]); (7) whereby work-related chronic stress (such as work overload, work discontent and pressure to succeed) seemed to have the strongest negative impact on the BFS/BFA.

Our expanded BeCS-14 subgroup “cognition” consisting of 147 healthy, educated, middle-class men and women had an average mean cognitive performance level. According to the IGD, the age-adjusted mean performance percentile rank was 52.9 ± 27.7 (range 0–100) and was therefore comparable with our previous study (52.4 ± 27.9) [6]. The results of the BFS cognitive-mental function subdomain and IGD were better in younger or better educated individuals, respectively, whereas there were no significant differences in respect to sex. These findings also support the results of our previous investigations [6].

On average, the bio-functional age of the participants was about 8-year equivalents below their chronological age. The age–BFA index was therefore comparable to our previous study with an age–BFA index of 9-year equivalents [6].

The correlation analysis between the BFS cognitive-mental function subdomain and the IGD showed a significant inverse correlation for total IGD and all IGD subdomains with nine BFS items: optical reaction time (M1), optical pursuing reaction time (M2), verbal reaction time (M4), cognitive reaction time (M5), cognitive switching capability (M6), strategic thinking (M9), changeover capability (M12), Stroop test (M4–M6) and Stepping-stone-maze test (M9–M12). All other BFS cognitive-mental function subdomain items (except for acoustical reaction time (M3)) significantly negatively correlated with (almost) all IGD domains, but verbal memory (VeM) and delayed recall (DR). We can therefore confirm the results of the previous study, which indicated that the ICF-based BFS/BFA assessment tool meets the requirements of the EIP-AHA [6].

When the correlation analysis was performed for three age-based subgroups (≤ 25 years, 26–49 years and ≥ 50 years), we found differences in correlation strength. For subjects aged ≤ 25 years, the correlation between BFS and IGD was weakest with five BFS subdomains being significantly correlated to IGD subdomains, while for subjects aged 26–49 years we found seven BFS subdomains, and for subjects aged ≥ 50 years ten BFS subdomains showing significant correlations with IGD subdomains, respectively. Overall, however, the significantly correlating subdomains were identical with those of the total study population. We can therefore assume, that the BFS cognitive-mental function subdomain is most accurate for the assessment of cognition of middle-aged or older people.

When compared to the mean values (T50) of the TICS reference population, the BeCS-14 subgroup “cognition” showed a similar chronic stress level overall (SSCS) and in almost all subdomains and is therefore comparable to our previous study population [7].

TICS-SSCS and all TICS subdomains were negatively correlated to the difference between chronological and bio-functional age (age–BFA index). Thus, an association of higher chronic stress exposure with a pro-aging state or less vitality (referring to our previous study [7]) can be suspected.

The correlation analysis between the TICS-Screening Scale for Chronic Stress (TICS-SSCS), which provides a global measure of chronic stress exposure, and various BFS cognitive-mental function subdomain items did not show significant correlations [7]. Importantly, the TICS-SSCS does not represent all TICS subdomains (only chronic worrying, lack of social recognition, excessive demands at work, work discontent and social overload). To investigate the effects of different aspects of chronic stress exposure in more detail, we performed single correlation analyses for TICS subdomains. Interestingly, several significant negative correlations between BFS cognitive-mental function subdomain items and three TICS subdomains were found. Work overload, work discontent and pressure to succeed—mainly work-related aspects of chronic stress exposure—showed the greatest negative correlation and thus may be suspected to have the biggest negative impact on mental-cognitive function. Work overload and pressure to succeed refer to stress resulting from high demands [10], which seems, among other occupational stress, to have a strong negative effect on the mental-cognitive BFS/BFA. In particular, age group 2 (26–49 years) and 3 (≥ 50 years), presenting significantly higher percentage of employees, showed a significantly higher level of chronic stress exposure than age group 1 (≤ 25 years). In contrast, social stress such as lack of social recognition, social overload, social isolation or social tensions did not seem to have an impact on BFS/BFA.

In the current study, the associations between work-related stress and cognition did not extend to all measured domains, but were specific to a few mental-cognitive (BFS) domains. In detail, participants with a higher level of work-related stress showed slower verbal and cognitive reaction time (M4, M5), worse cognitive switching capability (M6) and strategic thinking (M9), respectively. The effects of work-related stress on (late-life) cognition have been investigated in various studies before. Higher levels of (midlife) work-related stress were found to be associated with worse verbal learning and memory, but not visual memory [20], poorer episodic memory at retirement and more decline after retirement [21] and poorer performance in global cognition and processing speed [22]. As described before, different assessment tools of (work-related) stress and cognitive performance across the studies are a possible explanation for these discrepancies between the cognitive domains mostly concerned with occupational stress, as described before [22]. Further, associations between work-related stress and a higher risk of mild cognitive impairment, dementia and Alzheimer’s disease later in life were described [23–26]. Particularly, the combination of high job demands and low job control seems to play an important role when examining the impact of work-related stress on mental-cognitive function [27], as we were able to observe with our investigations.

Argued otherwise, findings demonstrated that cognitive abilities are an important personal resource that might protect individuals against the negative impacts of work-related stress and negative affect, whereby cognition might play a more valuable role for older than younger workers [28]. High levels of mental work demands, occupational complexity or job control were found to be prospectively associated with higher levels of cognitive function in midlife or late life [29] and a reduced risk for dementia [30]. Finally, the individual characteristic of reactivity to stress seems to be an important factor in the extent to which work-related stress has an effect on late-life cognition [25].

Our study clearly had some limitations, which have been described before [7]. Particular mention should be made of recruitment bias that cannot be ruled out and lack of randomization of participants.

In conclusion, our study confirms the results of our previous study [6] showing that the validated BFS/BFA assessment tool and its cognitive-mental function subdomain follow EIP-AHA requirements as it correlated well with the validated questionnaire for cognition assessment, the IGD. Further, we could demonstrate that chronic stress exposure, especially work-related stress, significantly negatively correlated with the BFS cognitive-mental function subdomain, indicating that higher levels of work-related stress may be associated with poorer mental-cognitive performance and a pro-aging state underlining our previous investigations [7]. These investigations could suggest that cognitive impairments can be reduced by stress management interventions which aim to reduce work-related stress. Future studies are needed to confirm this interpretation.

## Supplementary Information

Below is the link to the electronic supplementary material.

Supplementary file1 (DOCX 48 KB)

## Data Availability

Data are not available, as further publications from the data set are in preparation.

## Abbreviations

BeCS-14: Bern Cohort Study 2014
BFA: Bio-functional age
BFS: Bio-functional status
DR: Delayed recall
EIP-AHA: European Innovation Partnership on Active and Healthy Ageing
ICF: International Classification of Functioning, Disability and Health
IGD: Inventar zur Gedächtnisdiagnostik
L: Learning
NCD: Non-communicable disease
LeCS-84: Leipzig Cohort Study 1984
SSCS: Standardised Screening Scale for Chronic Stress (part of TICS)
SSPS: Statistical package for social sciences
ST: Short-term memory
T: Total memory
TICS: Trier Inventory for the assessment of Chronic Stress
WHO: World Health Organization
WM: Working memory
VeM: Verbal memory
ViM: Visual memory
