# Supplementary material for: Impact of chronic stress exposure on cognitive performance incorporating the active and healthy aging (AHA) concept within the cross-sectional Bern Cohort Study 2014 (BeCS-14)
Source: Arch Gynecol Obstet. 2021 Nov 6;305(4):1021–32. doi: 10.1007/s00404-021-06289-z (PMC8967732; doi:10.1007/s00404-021-06289-z)
Supplement: Supplementary file 1 — Supplementary file1 (DOCX 48 KB) [file 404_2021_6289_MOESM1_ESM.docx]

Supplementary table 1: Characteristics of the cohort

|  | Total  (n= 147) | age <= 25 years  (n= 45) | age 26-49 years  (n= 56) | age >= 50 years  (n= 44) | Exact Fisher-test  (exact significance, 2-sided) |
| --- | --- | --- | --- | --- | --- |
| 1) age  MW (+/-SD) | 38.0 (+/-15.3) | 22.7 (+/-1.9) | 34.4 (+/-7.7) | 57.9 (+/-6.1) | -- |
| 2) sex  M = male  W = female | M: 55 (37,4%)  W: 92 (62.6%) | M: 27 (60.0%)  W: 18 (40.0%) | M: 33 (57.9%)  W: 24 (42.1%) | M: 32 (71.1%)  W: 13 (28.9%) | 2.070 (0.365) |
| 3) highest education level  1: Matura  2: university /ETH / FH  3: others | 1: 33 (22.4%)  2: 46 (31.3%)  3: 68 (46.3%) | 1: 18 (40%)  2: 11 (24.4%)  3: 16 (35.6%) | 1: 9 (16.0%)  2: 27 (48.3%)  3: 20 (35.7%) | 1: 6 (13.6%)  2: 8 (18.2%)  3: 30 (68.2) | 29.96 (0.001)* |
| 4) job status  1: leading position  2: employee  3: internship / Lehrling  4: student  5: unemployed | 1: 23 (16.1%)  2: 78 (54.5%)  3: 5 (3.5%)  4: 32 (22.4%)  5: 5 (3.5%) | 1: 2 (4.4%)  2: 14 (31.1%)  3: 5 (11.1%)  4: 22 (48.9%)  5: 2 (4.4%) | 1: 9 (16.1%)  2: 37 (66.1%)  3: 0 (0.0%)  4: 10 (17.9%)  5: 0 (0.0%) | 1: 12 (28.6%)  2: 27 (64.3%)  3: 0 (0.0%)  4: 0 (0.0%)  5: 3 (7.1%) | 53.09 (<0.001)* |
| 5) monthly gross income  1: <=5000 CHF  2: 5000 -10’000CHF  3: >10'000 CHF  4: no income | 1: 72 (50.0%)  2: 55 (38.2%)  3: 5 (3.5%)  4: 12 (8.3%) | 1: 33 (73.3%)  2: 4 (8.9%)  3: 0 (0.0%)  4: 8 (17.8%) | 1: 21 (37.5%)  2: 27 (48.2%)  3: 4 (7.1%)  4: 4 (7.1%) | 1: 18 (41.9%)  2: 24 (55.8%)  3: 1 (2.3%)  4: 0 (0.0%) | 36.661 (<0.001)* |
| 6) alcohol consumption (frequency)  1 = never  2 = < 1x / month  3 = 2-4x / month  4 = 2-3x / week  5= 4x / week  6 = > 4x / week | 1: 15 (10.2%)  2: 14 (9.5%)  3: 65 (44.2%)  4: 35 (23.8%)  5: 11 (7.5%)  6: 7 (4.6%) | 1: 5 (11.1%)  2: 4 (8.9%)  3: 23 (51.5%)  4: 8 (17.8%)  5: 3 (6.7%)  6: 2 (4.4%) | 1: 6 (10.5%)  2: 3 (5.3%)  3: 27 (47.4%) 4: 13 (22.8%)  5: 4 (7.0%)  6: 4 (7.0%) | 1: 4 (8.9%)  2: 7 (15.6%)  3: 15 (33.3%)  4: 14 (31.1%)  5: 4 (8.9%)  6: 1 (2.2%) | 7.812 (0.657) |
| 7) alkohol consumption (quantity)  1 = none  2 = 1 glass / day  3 = >=2 glass / day | 1: 90 (61.2%)  2: 34 (23.1%)  3: 23 (15.7%) | 1: 29 (64.4%)  2: 9 (20.0%)  3: 7 (15.6%) | 1: 36 (63.2%)  2: 12 (21.1%)  3: 9 (15.7%) | 1: 25 (55.6%)  2: 13 (28.9%)  3: 7 (15.5%) | 8.884 (0.742) |
| 8) smoking  1 = non-smoker  2 = ex-smoker  3 = smoker | 1: 94 (63.9%)  2: 32 (21.8%)  3: 21 (14.3%) | 1: 35 (77.8%)  2: 5 (11.1%)  3: 5 (11.1%) | 1: 36 (63.2%)  2: 11 (19.3%)  3: 10 (17.5%) | 1: 23 (51.1%)  2: 16 (35.6%)  3: 6 (13.3%) | 13.940 (0.205) |
| 9) physical activity (till sweating)  1= <1x / week  2 = 1-2x/ week  3 = >2x / week | 1: 36 (24.5%)  2: 49 (33.3%)  3: 62 (42.2%) | 1: 12 (26.7%)  2: 12 (26.7%)  3: 21 (46.7%) | 1: 13 (22.8%)  2: 16 (28.1%)  3: 28 (49.1%) | 1: 11 (24.4%)  2: 21 (46.7%)  3: 13 (28.9%) | 14.860 (0.041)* |
| 10) sleep duration  1 = < 5h  2 = 6h  3 = 7h  4 = 8h  5 = > 9h | 1: 3 (2.0%)  2: 38 (25.9%)  3: 73 (49.7%)  4: 33 (22.4%)  5: 0 (0.0%) | 1: 0 (0.0%)  2: 12 (26.7%)  3: 22 (48.9%)  4: 11 (24.4%)  5: 0(0.0%) | 1: 1 (1.8%)  2: 16 (28.1%)  3: 27 (47.4%)  4: 13 (22.8%)  5: 0 (0.0%) | 1: 2 (4.4%)  2: 10 (22.2%)  3: 24 (53.3%)  4: 9 (20.0%) | 2.667 (0.895) |
| 11) high blood pressure  1 = no  2 = yes | 1: 129 (92.8%)  2: 10 (7.2%) | 1: 41 (95.3%)  2: 2 (4.7%) | 1: 53 (94.6%)  2: 3 (5.4%) | 1: 35 (87.5%)  2: 5 (12.5%) | 2.149 (0.388) |
| 12) stroke  1 = no  2 = yes | 1: 142 (99.3%)  2: 1 (0.7%) | 1: 44 (100.0%)  2: 0 (0.0%) | 1: 56 (100.0%)  2: 0 (0.0%) | 1: 42 (97.7%)  2: 1 (2.3%) | 2.059 (0.301) |
| 13) myocardial infarction  1 = no  2 = yes | 1: 137 (99.3%)  2: 1 (0.7%) | 1: 43 (10.00%)  2 = 0 (0.0%) | 1: 53 (100.0%)  2= 0 (0.0%) | 1: 41 (97.6%)  2: 1 (2.4%) | 2.031 (0.304) |
| 14) thrombosis  1 = no  2 = yes, currently  3 = yes, in the past | 1: 138 (93.9%)  2: 2 (1.4%)  3: 3 (2.0%) | 1: 43 (97.7%)  2: 1(2.3%)  3: 0 (0.0%) | 1: 55 (98.2%)  2: 1 (1.8%)  3: 0 (0.0%) | 1: 40 (93.0%)  2: 0 (0.0%)  3: 3 (7.0%) | 5.829 (0.058) |
| 15) pulmonary embolism  1 = no  2 = yes, currently  3 = yes, in the past | 1: 142 (96.6%)  2: 0 (0.0%)  3: 0 (0.0%) | 1: 44 (100%)  2: 0 (0.0%)  3: 0 (0.0%) | 1: 57 (100.0%)  2: 0 (0.0%)  3: 0 (0.0%) | 1: 41 (100.0%)  2: 0 (0.0%)  3: 0 (0.0%) | -- |
| 16) asthma  1 = no  2 = yes, currently  3 = yes, in the past | 1: 126 (89.4%)  2: 5 (3.5%)  3: 10 (7.1%) | 1: 37 (86.0%)  2: 1 (2.3%)  3: 5 (11.6%) | 1: 49 (89.1%)  2: 2 (3.6%)  3: 4 (7.3%) | 1: 40 (93.0%)  2: 2 (4.7%)  3: 1 (2.3%) | 3.169 (0.533) |
| 17) sleep apnea syndrome  1 = no  2 = yes, currently  3 = yes, in the past | 1: 142 (97.9%)  2: 2 (1.4%)  3: 1 (0.7%) | 1: 45 (100.0%)  2: 0 (0.0%)  3: 0 (0.0%) | 1: 57 (100.0%)  2: 0 (0.0%)  3: 0 (0.0%) | 1: 40 (93.0%)  2: 2 (4.7%)  3: 1 (2.3%) | 5.336 (0.025)* |
| 18) liver / gall bladder diseases  1 = no  2 = yes, currently  3 = yes, in the past | 1: 140 (96.6%)  2: 1 (0.7%)  3: 4 (2.7%) | 1: 44 (97.8%)  2: 0 (0.0%)  3: 1 (2.2%) | 1: 55 (96.5%)  2: 0 (0.0%)  3: 2 (3.5%) | 1: 41 (95.3%)  2: 1 (2.3%)  3: 1 (2.3%) | 2.495 (0.823) |
| 19) gastrointestinal diseases  1 = no  2 = yes, currently  3 = yes, in the past | 1: 120 (85.7%)  2: 9 (6.4%)  3: 11 (7.9%) | 1: 40 (90.9%)  2: 2 (4.5%)  3: 2 (4.5%) | 1: 48 (85.7%)  2: 4 (7.1%)  3: 4 (7.1%) | 1: 32 (80.0%)  2: 3 (7.5%)  3: 5 (12.5%) | 2.394 (0.689) |
| 20) kidney / ureteral diseases  1 = no  2 = yes, currently  3 = yes, in the past | 1: 133 (93.7%)  2: 2 (1.4%)  3: 7 (4.9%) | 1: 42 (95.5%)  2: 0 (0.0%)  3: 2 (4.5%) | 1: 53 (93.0%)  2: 0 (0.0%)  3: 4 (7.0%) | 1: 38 (92.7%)  2: 2 (4.9%)  3: 1 (2.4%) | 4.182 (0.308) |
| 21) diabetes mellitus  1: no  2: yes, currently | 1: 141 (99.3%)  2: 1 (0.7%) | 1: 44 (100.0%)  2: 0 (0.0%) | 1: 56 (100.0%)  2: 0 (0.0%) | 1: 41 (97.6%)  2: 1 (2.4%) | 2.095 (0.296) |
| 22) thyroid disorder  1 = no  2 = yes, currently  3 = yes, in the past | 1: 137 (95,8%)  2: 5 (3.5%)  3: 1 (0.7%) | 1: 42 (97.7%)  2: 1 (2.3%)  3: 0 (0.0%) | 1: 53 (94.6%)  2: 2 (3.6%)  3: 1 (1.8%) | 1: 42 (95.5%)  2: 2 (4.5%)  3: 0 (0.0%) | 2.034 (1.000) |
| 23) anaemia  1 = no  2 = yes, currently  3 = yes, in the past | 1: 129 (89.6%)  2: 5 (3.5%)  3: 10 (6.9%) | 1: 41 (93.2%)  2: 2 (4.5%)  3: 1 (2.3%) | 1: 48 (84.2%)  2: 3 (5.3%)  3: 6 (10.5%) | 1: 40 (93.0%)  2: 0 (0.0%)  3: 3 (7.0%) | 4.764 (0.278) |
| 24) dyslipidemia  1 = no  2 = yes, currently  3 = yes, in the past | 1: 124 (88.6%)  2: 12 (8.6%)  3: 4 (2.9%) | 1: 41 (97.6%)  2: 1 (2.4%)  3: 0 (0.0%) | 1: 50 (92.6%)  2: 1 (1.9%)  3: 3 (5.6%) | 1: 33 (75.0%)  2: 10 (22.7%)  3: 1 (2.3%) | 15.904 (<0.001)* |
| 25) muscular diseases  1 = no  2 = yes, currently  3 = yes, in the past | 1: 144 (99.3%)  2: 1 (0.7%)  3: 0 (0.0%) | 1: 45 (100.0%)  2: 0 (0.0%)  3: 0 (0.0%) | 1: 57 (100.0%)  2: 0 (0.0%)  3: 0 (0.0%) | 1: 42 (97.7%)  2: 1 (2.3%)  3: 0 (0.0%) | 2.088 (0.297) |
| 26) cancer  1 = no  2 = yes, currently  3 = yes, in the past | 1: 132 (96.4%)  2: 2 (1.5%)  3: 3 (2.2%) | 1: 44 (100.0%)  2: 0 (0.0%)  3: 0 (0.0%) | 1: 53 (100.0%)  2: 0 (0.0%)  3: 0 (0.0%) | 1: 35 (87.5%)  2: 2 (5.0%)  3: 3 (7.5%) | 8.456 (0.002)* |
| 27) depression  1 = no  2 = yes, currently  3 = yes, in the past | 1: 121 (85.5%)  2: 2 (1.4%)  3: 18 (12.8%) | 1: 40 (93.0%)  2: 1 (2.3%)  3: 2 (4.7%) | 1: 46 (82.1%)  2: 1 (1.8%)  3: 9 (16.1%) | 1: 35 (83.3%)  2: 0 (0.0%)  3: 7 (16.7%) | 4.920 (0.224) |
| 28) rheumatoid arthritis  1: no  2: yes, currently | 1: 142 (99.3%)  2: 1 (0.7%) | 1: 45 (100.0%)  2: 0 (0.0%) | 1: 55 (100.0%)  2: 0 (0.0%) | 1: 42 (97.7%)  2: 1 (2.3%) | 2.055 (0.301) |
| 29) arthrosis  1 = no  2 = yes, currently  3 = yes, in the past | 1: 126 (90.0%)  2: 12 (8.6%)  3: 2 (1.4%) | 1: 42 (97.9%)  2: 1 (2.3%)  3: 0 (0.0%) | 1: 52 (92.9%)  2: 3 (5.4%)  3: 1 (1.8%) | 1: 32 (78.0%)  2: 8 (19.5%)  3: 1 (2.4%) | 9.148 (0.022)* |
| 30) osteoporosis  1 = no  2 = yes, currently  3 = yes, in the past | 1: 134 (95.7%)  2: 5 (3.6%)  3: 1 (0.7%) | 1: 44 (97.8%)  2: 0 (0.0%)  3: 1 (2.2%) | 1: 56 (100.0%)  2: 0 (0.0%)  3: 0 (0.0%) | 1: 34 (87.2%)  2: 5 (12.8%)  3: 0 (0.0%) | 11.543 (0.001)* |

*: the correlation is significant at the level 0.05 (2-sided)

Supplementary table 2: Impact of age, education and sex on BFS subdomains

| Cognitive and mental function (BFS) | Kruskal-Wallis test /  Mann-Whitney U test  (asympt. significance);  comparison for age subgroups | Kruskal-Wallis test  (asympt. significance);  comparison for education subgroups | Mann-Whitney U test  (asympt. significance);  comparison for sex subgroups |
| --- | --- | --- | --- |
| M1^1^ | 7.66 (0.022)*  1 vs. 2: 0.038*  1 vs. 3: 0.337  2 vs. 3: 0.015* | 10.05 (0.074) | 2.79 (0.095) |
| M2^2^ | 32.59 (<0.001)*  1 vs. 2: 0.038*  2 vs. 3: <0.001* | 7.27 (0.202) | 16.37 (<0.001)* |
| M3^3^ | 7.59 (0.023)*  1 vs. 2: 0.217  1 vs. 3: 0.005*  2 vs. 3: 0.113 | 6.85 (0.232) | 1.30 (0.254) |
| M4^4^ | 30.32 (<0.001)*  1 vs. 2: 0.871  1 vs. 3: <0.001* | 17.14 (0.004)* | 1.71 (0.191) |
| M5^5^ | 23.83 (<0.001)*  1 vs. 2: 0.557  1 vs. 3: <0.001* | 11.55 (0.042)* | 0.08 (0.777) |
| M6^6^ | 47.36 (<0.001)*  2 vs. 3: <0.001* | 34.74 (<0.001)* | 0.15 (0.702) |
| M7^7^ | 28.35 (<0.001)*  2 vs. 3: <0.001* | 10.06 (0.074) | 0.25 (0.619) |
| M8^8^ | 6.58 (0.037)*  1 vs. 2: 0.059  1 vs. 3: 0.004*  2 vs. 3: 0.231 | 12.82 (0.025)* | 1.18 (0.277) |
| M9^9^ | 26.58 (<0.001)*  2 vs. 3: <0.001 | 17.04 (0.004)* | 1.83 (0.176) |
| M10^10^ | 6.59 (0.037)*  1 vs. 3:  2 vs. 3: 0.092 | 3.86 (0.570) | 0.07 (0.794) |
| M11^11^ | 15.08 (0.001)*  2 vs. 3: 0.002* | 9.36 (0.095) | 1.29 (0.256) |
| M12^12^ | 20.42 (<0.001)*  2 vs. 3: <0.001 | 10.78 (0.056) | 1.37 (0.243) |

1: optical reaction time, 2: pursuing reaction time, 3: acoustical reaction time, 4: verbal reaction time, 5: cognitive reaction time, 6: cognitive switching capability, 7: ability to concentrate (time), 8: ability to concentrate (mistakes), 9: strategic thinking, 10: memory performance, 11: orientation capability, 12: changeover capability, *: the correlation is significant at the level 0.05 (2-sided)

Supplementary table 3: Cognitive performance assessed by IGD

| IGD subtest /  IGD subdomain | Total (n = 147)  Mean (+/- SD) | <=25 (n = 45)  Mean (+/- SD) | 26 – 49 (n = 56)  Mean (+/- SD) | >= 50 (n = 44)  Mean (+/- SD) |
| --- | --- | --- | --- | --- |
| Total^1^ | 182.58 (28.56) | 191.91 (18.60) | 189.93 (23.08) | 163.97 (34.20) |
| A1^2^ | 20.22 (5.77) | 22.04 (4.48) | 20.63 (4.65) | 17.87 (7.32) |
| A2^3^ | 17.10 (3.75) | 18.00 (3.16) | 17.72 (3.90) | 15.42 (3.61) |
| A3^4^ | 16.35 (3.22) | 17.22 (2.40) | 16.84 (3.20) | 14.84 (3.49) |
| A4^5^ | 15.89 (4.39) | 18.11 (2.93) | 16.25 (4.21) | 13.22 (4.51) |
| A5^6^ | 17.12 (5.85) | 19.36 (4.82) | 18.47 (4.84) | 13.16 (6.08) |
| A6^7^ | 15.09 (2.79) | 16.00 (2.76) | 15.26 (2.63) | 13.96 (2.68) |
| A7^8^ | 15.84 (4.46) | 17.24 (3.39) | 16.60 (3.93) | 13.47 (5.12) |
| A8^9^ | 19.73 (3.42) | 20.67 (1.83) | 20.42 (1.65) | 17.93 (5.22) |
| A9^10^ | 12.84 (3.46) | 14.00 (2.09) | 12.82 (3.62) | 11.69 (4.01) |
| A10^11^ | 13.14 (4.31) | 13.82 (3.98) | 13.70 (4.03) | 11.76 (4.73) |
| A11^12^ | 18.04 (3.58) | 19.11 (1.68) | 19.12 (2.20) | 15.60 (4.97) |
| A12^13^ | 8.59 (5.34) | 8.93 (5.18) | 10.00 (5.54) | 6.47 (4.65) |
| ST/WM^14^ | 50.34 (10.40) | 55.02 (7.47) | 52.74 (8.93) | 42.61 (10.51) |
| L^15^ | 50.66 (8.09) | 53.91 (5.60) | 52.28 (5.51) | 45.36 (10.21) |
| VeM^16^ | 57.42 (9.15) | 61.04 (6.80) | 58.63 (7.86) | 52.24 (10.48) |
| ViM^17^ | 49.77 (10.65) | 54.47 (6.28) | 51.96 (8.70) | 42.29 (12.42) |
| DR^18^ | 44.02 (8.11) | 46.93 (5.06) | 45.65 (6.49) | 39.04 (10.09) |

1: total memory (A1-A12), 2: prospective memory, 3: forward digit span, 4: verbal working memory, 5: visual working memory, 6: executive function, 7: verbal learning, 8: visual learning, 9: pair association, 10: delayed recognition: wordlist, 11: delayed recognition: text, 12: delayed recognition: figures, 13: priming, 14: short term memory/working memory, 15: learning, 16: verbal memory, 17: visual memory, 18: delayed recall

Supplementary table 4: Impact of age, education and sex on IGD subdomains

| IGD subtest /  IGD subdomain | Kruskal-Wallis test / Mann-Whitney U test  (asympt. significance);  comparison for age subgroups | Kruskal-Wallis test  (asympt. significance);  comparison for education subgroups | Mann-Whitney U test  (asympt. significance);  comparison for sex subgroups |
| --- | --- | --- | --- |
| Total^1^ | 24.44 (<0.001)*  2 vs. 3: <0.001* | 49.02 (<0.001)* | 0.39 (0.531) |
| A1^2^ | 12.08 (0.002)*  2 vs. 3: 0.077* | 8.10 (.151) | 0.67 (0.414) |
| A2^3^ | 12.51 (0.002)*  2 vs. 3: 0.005* | 22.02 (0.001)* | 0.97 (0.321) |
| A3^4^ | 15.98 (<0.001)*  2 vs. 3: 0.001 | 36.68 (<0.001)* | 0.04 (0.848) |
| A4^5^ | 27.27 (<0.001)*  2 vs. 3: 0.001 | 30.71 (<0.001)* | 1.07 (0.300) |
| A5^6^ | 26.94 (<0.001)*  2 vs. 3: <0.001* | 38.06 (<0.001)* | 2.38 (0.123) |
| A6^7^ | 13.57 (0.001)*  2 vs. 3: 0.021* | 13.17 (0.022)* | 0.96 (0.327) |
| A7^8^ | 16.80 (<0.001)*  2 vs. 3: 0.001* | 40.77 (<0.001)* | 2.39 (0.122) |
| A8^9^ | 17.96 (<0.001)*  2 vs. 3: 0.003* | 22.84 (<0.001)* | 0.92 (0.337) |
| A9^10^ | 10.87 (0.004)*  2 vs. 3: 0.061  1 vs. 3: 0.001* | 13.56 (0.019)* | 2.51 (0.113) |
| A10^11^ | 5.69 (0.058)  2 vs. 3: 0.035* | 22.99 (<0.001)* | 4.78 (0.029)* |
| A11^12^ | 32.63 (<0.001)*  2 vs. 3: <0.001*  1 vs. 3: <0.001* | 20.97 (0.001)* | 2.67 (0.102) |
| A12^13^ | 13.40 (0.001)*  2 vs. 3: <0.001*  1 vs. 3: <0.001* | 14.61 (0.012)* | 0.70 (0.402) |
| ST/WM^14^ | 35.05 (<0.001)*  2 vs. 3: <0.001* | 52.00 (<0.001)* | 1.85 (0.173) |
| L^15^ | 24.26 (<0.001)*  2 vs. 3: <0.001* | 44.98 (<0.001)* | 0.10 (0.758) |
| VeM^16^ | 19.81 (<0.001)*  2 vs. 3: 0.002* | 37.47 (<0.001)* | 0.07 (0.791) |
| ViM^17^ | 31.86 (<0.001)*  2 vs. 3: <0.001* | 39.48 (<0.001)* | 2.92 (0.087) |
| DR^18^ | 20.04 (<0.001)*  2 vs. 3: <0.001* | 31.33 (<0.001)* | 2.64 (0.104) |

1: total memory (A1-A12), 2: prospective memory, 3: forward digit span, 4: verbal working memory, 5: visual working memory, 6: executive function, 7: verbal learning, 8: visual learning, 9: pair association, 10: delayed recognition: wordlist, 11: delayed recognition: text, 12: delayed recognition: figures, 13: priming, 14: short term memory/working memory, 15: learning, 16: verbal memory, 17: visual memory, 18: delayed recall, *: the correlation is significant at the level 0.05 (2-sided)

Supplementary table 5: Correlation analysis between the mental-cognitive BFS and a validated cognitive performance test battery (IGD) with age-based subgroup analysis

| IGD subdomains | Age (years) | Correlation coeficient (r)  p-value (p) | Cognitive and mental function (BFS) | | | | | | | | | | | | | | |
| --- | --- | --- | --- | --- | --- | --- | --- | --- | --- | --- | --- | --- | --- | --- | --- | --- | --- |
|  |  |  | M1^1^ | M2^2^ | M3^3^ | M4^4^ | M5^5^ | M6^6^ | M7^7^ | M8^8^ | M9^9^ | M10^10^ | M11^11^ | M12^12^ | Stroop test (M4-M6) | Landolt test (M7-M8) | Stepping-stone-maze test (M9-M12) |
| Total^13^ | Total | r  p | -0.442**  <0.001 | -0.440**  <0.001 | -0.940  0.261 | -0.418**  <0.001 | -0-516**  <0.001 | -0.594**  <0.001 | -0.197*  0.018 | -0.191*  0.022 | -0.524**  <0.001 | -0.041  0.624 | -0.090  0.281 | -0.570**  <0.001 | -0.617**  <0.001 | -0.205*  0.014 | -0.415**  <0.001 |
|  | <= 25 | r  p | -0.005  0.977 | 0.092  0.56 | 0.052  0.744 | -0.084  0.595 | -0.178  0.261 | -0.387*  0.011 | -0.189  0.231 | -0.072  0.651 | -0.403**  0.008 | 0.166  0.294 | 0.270  0.084 | -0.448**  0.003 | 0.331*  0.045 | -0.194  0.218 | -0.273  0.080 |
|  | 26-49 | r  p | -0.188  0.162 | -0.082  0.544 | -0.189  0.159 | -0.057  0.674 | -0.189  0.158 | -0.261  0.050 | 0.072  0.593 | -0.075  0.577 | -0.211  0.115 | -0.135  0.316 | -0.030  0.822 | -0.235  0.078 | -0.239  0.074 | 0.069  0.608 | -0.168  0.211 |
|  | >= 50 | r  p | -0.619**  <0.001 | -0.538**  <0.001 | -0.321*  0.031 | -0.415**  0.005 | -0.564**  <0.001 | -0.593**  <0.001 | -0.033  0.827 | -0.182  0.233 | -0.549**  <0.001 | 0.125  0.412 | -0.303  0.845 | -0.595**  <0.001 | -0.638**  <0.001 | -0.042  0.786 | -0.451**  0.002 |
| ST/WM^14^ | Total | r  p | -0.231**  0.005 | -0.430**  <0.001 | 0.213*  0.010 | -0.451**  <0.001 | -0.494**  <0.001 | -0.576**  <0.001 | -0.278**  0.001 | -0.177*  0.034 | -0.484**  <0.001 | -0.187*  0.025 | -0.202*  0.015 | -0.438**  <0.001 | -0.604**  <0.001 | -0.287**  <0.001 | -0.438**  <0.001 |
|  | <= 25 | r  p | 0.185  0.242 | 0.228  0.147 | 0.147  0.352 | -0.171  0.279 | -0.237  0.131 | -0.311*  0.045 | -0.218  0.166 | -0.176  0.265 | -0.190  0.228 | -0.277  0.076 | 0.074  0.639 | -0.139  0.379 | -0.311*  0.034 | -0.288  0.146 | -0.232  0.139 |
|  | 26-49 | r  p | -0.003  0.981 | -0.358**  0.006 | 0.185  0.168 | -0.176  0.191 | -0.225  0.093 | -0.377**  0.004 | -0.083  0.539 | -0.109  0.421 | -0.380**  0.004 | -0.366**  0.005 | -0.252  0.059 | -0.196  0.143 | -0.353**  0.007 | -0.089  0.512 | -0.377**  0.004 |
|  | >= 50 | r  p | -0.371*  0.012 | -0.361*  0.015 | 0.047  0.757 | -0.390*  0.008 | -0.474**  0.001 | -0.554**  <0.001 | -0.036  0.815 | -0.036  0.816 | -0.396**  0.007 | 0.157  0.303 | 0.013  0.932 | -0.438**  0.003 | -0.588**  <0.001 | -0.038  0.806 | -0.308*  0.040 |
| L^15^ | Total | r  p | -0.205*  0.014 | -0.474**  <0.001 | 0.272**  0.001 | -0.351*  <0.001 | -0.431*  <0.001 | -0.499**  <0.001 | -0.249**  0.003 | -0.173*  0.038 | -0.488**  <0.001 | -0.204*  0.014 | -0.243**  0.003 | -0.425**  <0.001 | -0.518**  <0.001 | -0.257**  0.002 | -0.452**  <0.001 |
|  | <= 25 | r  p | 0.154  0.329 | 0.022  0.888 | 0.365*  0.018 | -0.214  0.174 | -0.212  0.178 | -0.309*  0.046 | -0.086  0.587 | -0.084  0.598 | -0.074  0.643 | 0.039  0.807 | 0.126  0.427 | -0.112  0.481 | -0.312*  0.044 | -0.091  0.567 | -0.031  0.844 |
|  | 26-49 | r  p | 0.186  0.165 | -0.202  0.132 | 0.325*  0.014 | -0.100  0.458 | -0.187  0.163 | -0.334*  0.011 | -0.174  0.195 | -0.046  0.733 | -0.375**  0.004 | -0.426**  0.001 | -0.229  0.087 | -0.114  0.399 | -0.296*  0.026 | -0.177  0.187 | -0.389**  0.003 |
|  | >= 50 | r  p | -0.350*  0.018 | -0.512**  <0.001 | 0.089  0.561 | -0.204  0.179 | -0.358*  0.016 | -0.392**  0.008 | -0.023  0.881 | -0.137  0.370 | -0.439**  0.003 | -0.041  0.787 | -0.223  0.142 | -0.409**  0.005 | -0.411**  0.005 | -0.029  0.850 | -0.414**  0.005 |
| VeM^16^ | Total | r  p | -0.188*  0.024 | -0.317**  <0.001 | 0.098  0.241 | -0.306**  <0.001 | -0.296**  <0.001 | -0.359**  <0.001 | -0.135  0.106 | -0.036  0.667 | -0.343**  <0.001 | -0.159  0.057 | -0.194*  0.020 | -0.284**  0.001 | -0.378**  <0.001 | -0.137  0.101 | -0.325**  <0.001 |
|  | <= 25 | r  p | 0.132  0.404 | 0.105  0.506 | 0.021  0.893 | -0.076  0.633 | -0.100  0.528 | -0.093  0.560 | -0.085  0.592 | 0.088  0.578 | 0.053  0.740 | -0.110  0.486 | -0.077  0.629 | 0.080  0.615 | -0.105  0.506 | -0.082  0.606 | 0.004  0.981 |
|  | 26-49 | r  p | 0.141  0.297 | -0.062  0.646 | 0.119  0.376 | -0.203  0.130 | -0.117  0.386 | -0.153  0.256 | -0.070  0.603 | 0.072  0.597 | -0.203  0.129 | -0.289*  0.029 | -0.178  0.184 | 0.011  0.938 | -0.183  0.173 | -0.068  0.618 | -0.239  0.073 |
|  | >= 50 | r  p | -0.375*  0.011 | -0.339*  0.023 | -0.093  0.542 | -0.156  0.305 | -0.194  0.203 | -0.274  0.069 | 0.156  0.307 | 0.016  0.919 | -0.297*  0.048 | 0.055  0.720 | -0.081  0.597 | -0.300*  0.046 | -0.280  0.063 | 0.158  0.300 | -0.255  0.091 |
| ViM^17^ | Total | r  p | -0.246**  0.003 | -0.482**  <0.001 | 0.302*  <0.001 | -0.347**  <0.001 | -0.463**  <0.001 | -0.572**  <0.001 | -0.218**  0.009 | -0.256**  0.002 | -0.488**  <0.001 | -0.188*  0.024 | -0.243**  0.003 | -0.425**  <0.001 | -0.581**  <0.001 | -0.230**  0.006 | -0.448**  <0.001 |
|  | <= 25 | r  p | 0.080  0.615 | 0.031  0.847 | 0.230  0.143 | -0.228  0.147 | -0.282  0.071 | -0.474**  0.002 | -0.173  0.274 | -0.282  0.070 | -0.321*  0.038 | 0.003  0.987 | 0.149  0.346 | -0.341*  0.027 | -0.446**  0.003 | -0.188  0.234 | -0.265  0.090 |
|  | 26-49 | r  p | 0.062  0.649 | -0.288*  0.030 | 0.325*  0.014 | -0.055  0.685 | -0.170  0.207 | -0.273*  0.040 | <0.001  0.997 | -0.191  0.115 | -0.296*  0.026 | -0.302*  0.023 | -0.238  0.074 | -0.092  0.498 | -0.240  0.072 | -0.009  0.946 | -0.306*  0.021 |
|  | >= 50 | r  p | -0.373*  0.012 | -0.460**  0.001 | 0.154  0.314 | -0.195  0.198 | -0.410**  0.005 | -0.518**  <0.001 | <0.001  0.998 | -0.156  0.305 | -0.419**  0.004 | -0.046  0.767 | -0.162  0.288 | -0.378*  0.010 | -0.523**  <0.001 | -0.006  0.976 | -0.390**  0.008 |
| DR^18^ | Total | r  p | -0.230**  0.006 | -0.395**  <0.001 | 0.149  0.074 | -0.287**  <0.001 | -0.333**  <0.001 | -0.425**  <0.001 | -0.075  0.374 | -0.116  0.116 | -0.331**  <0.001 | -0.024  0.773 | -0.145  0.083 | -0.322**  <0.001 | -0.434**  <0.001 | -0.080  0.343 | -0.275**  0.001 |
|  | <= 25 | r  p | 0.197  0.210 | 0.091  0.565 | -0.124  0.435 | 0.004  0.980 | 0.103  0.516 | 0.006  0.970 | -0.040  0.802 | -0.031  0.843 | 0.049  0.760 | -0.015  0.927 | -0.075  0.635 | 0.059  0.711 | 0.032  0.843 | -0.042  0.793 | 0.025  0.875 |
|  | 26-49 | r  p | 0.098  0.468 | -0.116  0.389 | 0.163  0.225 | -0.143  0.228 | -0.139  0.302 | -0.091  0.502 | 0.139  0.303 | 0.006  0.967 | 0.044  0.745 | -0.006  0.963 | -0.016  0.907 | 0.071  0.602 | -0.135  0.316 | 0.140  0.299 | 0.020  0.882 |
|  | >= 50 | r  p | 0.383**  0.009 | -0.423**  0.004 | 0.038  0.804 | -0.128  0.401 | -0.272  0.071 | -0.373*  0.012 | 0.143  0.347 | -0.080  0.600 | -0.357*  0.016 | 0.041  0.788 | -0.125  0.414 | -0.355*  0.024 | -0.372*  0.012 | 0.141  0.355 | -0.315*  0.035 |

1: optical reaction time, 2: pursuing reaction time, 3: acoustical reaction time, 4: verbal reaction time, 5: cognitive reaction time, 6: cognitive switching capability, 7: ability to concentrate (time), 8: ability to concentrate (mistakes), 9: strategic thinking, 10: memory performance, 11: orientation capability, 12: changeover capability, 13: total memory (A1-A12), 14: short term memory / working memory (A2-A5), 15: learning (A6-A8), 16: verbal memory (A3, A6, A9, A10), 17: visual memory (A4, A7, A11), 18: delayed recall (A9-A11), **: the correlation is significant at the level 0.01 (2-sided), *: the correlation is significant at the level 0.05 (2-sided)

Supplementary table 6: Impact of age, education and sex on TICS subdomains

| TICS subdomain | Kruskal-Wallis test  (asympt. significance); comparison for age subgroups | Kruskal-Wallis test  (asympt. significance); comparison for education subgroups | Mann-Whitney U test  (asympt. significance); comparison for sex subgroups |
| --- | --- | --- | --- |
| Chronic worrying | 0.41 (0.814) | 10.92 (0.053) | 2388.50 (0.643) |
| Excessive demands at work | 1.57 (0.456) | 12.97 (0.024)* | 2228.00 (0.489) |
| Work overload | 6.22 (0.045)*  1 vs. 2: 0.117  2 vs. 3: 0.018*  1 vs. 3: 0.296 | 10.19 (0.070) | 2010.00 (0.088) |
| Work discontent | 14.77 (0.001)*  1 vs. 2: 0.381  2 vs. 3: 0.003* | 23.09 (0.001)* | 2278.50 (0.364) |
| Pressure to perform | 15.66 (<0.001)*  1 vs. 2: 0.002*  1 vs. 3: 0.397 | 15.22 (0.009)* | 2412.50 (0.942) |
| Lack of social recognition | 4.45 (<0.001)*  1 vs. 2: 0.396  2 vs. 3: 0.043*  1 vs. 3: 0.180 | 3.84 (0.572) | 2476.50 (0.916) |
| Social overload | 10.05 (0.007)*  2 vs. 3: 0.630  1 vs. 3: 0.008* | 2.00 (0.849) | 2168.0 (0.175) |
| Social isolation | 3.17 (0.205) | 12.37 (0.030)* | 2487.50 (0.951) |
| Social tensions | 2.93 (0.231) | 4.12 (0.532) | 2306.00 (0.425) |
| Screening Scale (SSCS) | 1.36 (0.506) | 14.47 (0.013)* | 2189.00 (0.337) |

*: the correlation is significant at the level 0.05 (2-sided)

Supplementary table 7: Correlation analysis between the mental-cognitive BFS and chronic stress exposure (TICS) with age-based subgroup analysis

| TICS  subdomains | Age (years) | Correlation coeficient (r)  p-value (p) | Cognitive and mental function (BFS) | | | | | | | | | | | | | | |
| --- | --- | --- | --- | --- | --- | --- | --- | --- | --- | --- | --- | --- | --- | --- | --- | --- | --- |
|  |  |  | M1^1^ | M2^2^ | M3^3^ | M4^4^ | M5^5^ | M6^6^ | M7^7^ | M8^8^ | M9^9^ | M10^10^ | M11^11^ | M12^12^ | Stroop test (M4-M6) | Landolt test (M7-M8) | Stepping-stone-maze test (M9-M12) |
| Chronic worrying | Total | r  p | 0.203*  0.015 | 0.069  0.410 | 0.184*  0.028 | -0.038  0.655 | 0.021  0.801 | -0.012  0.882 | -0.079  0.350 | 0.144  0.086 | 0.006  0.939 | -0.033  0.692 | 0.015  0.863 | 0.018  0.833 | -0.012  0.890 | -0.073  0.386 | -0.001  0.988 |
|  | <= 25 | r  p | 0.030  0.853 | 0.078  0.623 | 0.052  0.746 | -0.039  0.807 | -0.081  0.612 | -0.149  0.347 | -0.230  0.142 | 0.307*  0.048 | -0.032  0.839 | 0.177  0.263 | 0.108  0.495 | -0.078  0.624 | -0.132  0.406 | -0.219  0.164 | 0.039  0.805 |
|  | 26-49 | r  p | 0.361**  0.006 | 0.035  0.798 | 0.329*  0.013 | 0.044  0.743 | 0.124  0.359 | -0.087  0.520 | -0.004  0.975 | 0.011  0.932 | -0.060  0.567 | -0.169  0.208 | -0.081  0.547 | 0.097  0.473 | -0.008  0.953 | -0.004  0.978 | -0.103  0.447 |
|  | >= 50 | r  p | 0.216  0.158 | 0.213  0.164 | -0.002  0.991 | -0.090  0.562 | 0.044  0.778 | 0.104  0.504 | -0.061  0.693 | 0.302*  0.046 | 0.147  0.342 | 0.249  0.103 | 0.220  0.152 | 0.061  0.694 | 0.082  0.596 | -0.049  0754 | 0.202  0.188 |
| Excessive demands at work | Total | r  p | 0.152  0.074 | -0.021  0.803 | 0.213*  0.012 | -0.090  0.289 | -0.015  0.856 | -0.083  0.332 | -0.035  0.686 | 0.104  0.223 | -0.078  0.359 | -0.33  0.702 | 0.018  0.837 | -0.076  0.373 | -0.081  0.342 | -0.030  0.722 | -0.064  0.456 |
|  | <= 25 | r  p | 0.249  0.112 | -0.117  0.462 | 0.116  0.464 | 0.136  0.389 | 0.148  0.349 | -0.002  0.990 | -0.069  0.662 | -0.427**  0.005 | 0.019  0.906 | 0.087  0.584 | -0.015  0.924 | 0.016  0.921 | 0.065  0.683 | -0.051  0.750 | 0.037  0.817 |
|  | 26-49 | r  p | 0.285*  0.037 | 0.118  0.397 | 0.356**  0.008 | 0.004  0.975 | 0.165  0.232 | 0.013  0.926 | 0.076  0.583 | 0.168  0.224 | -0.021  0.883 | -0.096  0.488 | -0.017  0.905 | 0.065  0.643 | 0.060  0.665 | 0.084  0.544 | -0.045  0.745 |
|  | >= 50 | r  p | 0.073  0.643 | 0.014  0.931 | -0.010  0.947 | -0.192  0.218 | -0.139  0.374 | -0.091  0.561 | -0.054  0.733 | -0.105  0.501 | -0.095  0.545 | 0.091  0.560 | 0.165  0.290 | -0.142  0.365 | -0.122  0.435 | -0.059  0.709 | -0.042  0.791 |
| Work overload | Total | r  p | -0.082  0.336 | -0.150  0.077 | 0.170*  0.045 | -0.176*  0.037 | -0.211*  0.012 | -0.233**  0.006 | -0.168*  0.047 | 0.093  0.274 | -0.127  0.135 | 0.011  0.897 | 0.015  0.858 | -0.149  0.078 | -0.246**  0.003 | -0.165  0.052 | -0.090  0.292 |
|  | <= 25 | r  p | 0.021  0.897 | 0.052  0.748 | 0.092  0.568 | -0.002  0.992 | -0.088  0.586 | -0.273  0.084 | -0.284  0.072 | 0.109  0.498 | -0.063  0.695 | -0.173  0.279 | 0.077  0.632 | -0.032  0.844 | -0.212  0.184 | -0.282  0.074 | -0.087  0.587 |
|  | 26-49 | r  p | 0.141  0.304 | -0.087  0.527 | 0.291*  0.031 | 0.007  0.960 | 0.006  0.963 | -0.073  0.598 | -0.039  0.779 | 0.207  0.129 | 0.043  0.756 | -0.027  0.846 | -0.037  0.790 | 0.129  0.349 | -0.044  0.751 | -0.029  0.831 | 0.009  0.947 |
|  | >= 50 | r  p | -0.177  0.250 | -0.164  0.288 | 0.015  0.923 | -0.239  0.118 | -0.297  0.051 | -0.247  0.106 | -0.129  0.404 | 0.022  0.889 | -0.171  0.267 | 0.125  0.419 | 0.128  0.406 | -0.229  0.135 | -0.283  0.062 | -0.129  0.403 | -0.106  0.495 |
| Work discontent | Total | r  p | 0.064  0.450 | -0.177*  0.034 | 0.174*  0.038 | -0.188*  0.024 | -0.127  0.130 | -0.175*  0.036 | -0.141  0.094 | 0.054  0.520 | -0.299**  0.006 | -0.125  0.136 | -0.132  0.115 | -0.186*  0.026 | -0.187*  0.025 | -0.139  0.098 | -0.222**  0.008 |
|  | <= 25 | r  p | 0.190  0.229 | 0.005  0.973 | 0.232  0.140 | 0.057  0.721 | 0.020  0.901 | -0.131  0.408 | -0.091  0.565 | 0.091  0.568 | -0.131  0.407 | 0.197  0.211 | 0.026  0.869 | 0.095  0.551 | -0.074  0.642 | -0.088  0.579 | 0.179  0.258 |
|  | 26-49 | r  p | 0.135  0.318 | -0.217  0.105 | 0.176  0.189 | 0.023  0.865 | 0.130  0.334 | -0.061  0.654 | 0.030  0.827 | 0.241  0.071 | -0.249  0.062 | -0.219  0.101 | -0.196  0.143 | -0.141  0.296 | 0.006  0.967 | 0.041  0.762 | -0.246  0.065 |
|  | >= 50 | r  p | 0.168  0.274 | 0.144  0.352 | -0.058  0.710 | -0.207  0.177 | -0.108  0.487 | 0.011  0.942 | -0.020  0.896 | -0.017  0.912 | -0.118  0.445 | 0.020  0.895 | 0.134  0.387 | -0.141  0.362 | 0.193  0.209 | -0.021  0.892 | 0.384*  0.011 |
| Pressure to perform | Total | r  p | -0.090  0.289 | -0.200*  0.018 | 0.086  0.310 | -0.240**  0.004 | -0.203*  0.016 | -0.274**  0.001 | -0.157  0.063 | 0.059  0.488 | -0.280**  0.001 | -0.064  0.449 | -0.064  0.449 | -0.076  0.368 | -0.286**  0.001 | -0.155  0.066 | -0.237**  0.005 |
|  | <= 25 | r  p | 0.187  0.243 | 0.087  0.589 | -0.031  0.846 | 0.383*  0.013 | 0.165  0.304 | -0.069  0.670 | -0.104  0.516 | 0.070  0.663 | -0.015  0.927 | -0.019  0.907 | 0.014  0.932 | -0.005  0.974 | 0.074  0.648 | -0.102  0.524 | -0.016  0.922 |
|  | 26-49 | r  p | 0.150  0.267 | -0.068  0.617 | 0.165  0.221 | -0.104  0.440 | 0.080  0.552 | -0.120  0.375 | -0.254  0.056 | 0.224  0.095 | -0.276  0.038 | -0.205  0.125 | -0.179  0.182 | -0.195  0.146 | -0.079  0.651 | -0.246  0.065 | -0.254  0.057 |
|  | >= 50 | r  p | -0.212  0.172 | -0.272  0.078 | -0.068  0.663 | -0.382*  0.012 | -0.385*  0.011 | -0.335*  0.028 | 0.121  0.441 | -0.122  0.436 | -0.266  0.084 | 0.169  0.279 | 0.135  0.388 | -0.351*  0.021 | -0.388*  0.010 | 0.117  0.457 | -0.178  0.253 |
| Lack of social recognition | Total | r  p | 0.026  0.760 | -0.120  0.154 | 0.113  0.178 | -0.058  0.492 | -0.136  0.105 | -0.108  0.200 | -0.053  0.528 | 0.011  0.895 | -0.080  0.340 | -0.026  0.758 | -0.039  0.758 | -0.039  0.643 | -0.117  0.165 | -0.053  0.530 | -0.072  0.391 |
|  | <= 25 | r  p | 0.073  0.648 | -0.027  0.865 | 0.007  0.965 | 0.082  0.604 | -0.021  0.893 | -0.174  0.271 | -0.027  0.867 | 0.086  0.587 | -0.004  0.982 | 0.086  0.587 | 0.076  0.633 | -0.024  0.881 | -0.109  0.493 | -0.023  0.885 | 0.035  0.824 |
|  | 26-49 | r  p | 0.120  0.375 | -0.071  0.599 | 0.149  0.270 | -0.013  0.922 | -0.101  0.455 | -0.074  0.583 | 0.137  0.308 | 0.087  0.519 | -0.088  0.517 | -0.134  0.321 | -0.148  0.271 | 0.045  0.739 | -0.081  0.547 | 0.142  0.291 | -0.119  0.379 |
|  | >= 50 | r  p | 0.049  0.754 | -0.079  0.611 | 0.043  0.780 | 0.029  0.850 | -0.073  0.637 | -0.022  0.890 | -0.173  0.260 | -0.103  0.507 | 0.016  0.919 | 0.183  0.234 | 0.192  0.211 | -0.059  0.702 | -0.025  0.871 | -0.179  0.244 | 0.074  0.634 |
| Social overload | Total | r  p | 0.072  0.394 | -0.062  0.462 | 0.007  0.930 | -0.016  0.852 | -0.038  0.655 | -0.045  0.594 | -0.002  0.979 | 0.107  0.204 | -0.082  0.331 | 0.022  0.794 | 0.080  0.343 | -0.127  0.130 | -0.044  0.600 | 0.002  0.979 | -0.043  0.607 |
|  | <= 25 | r  p | 0.296  0.057 | 0.143  0.365 | 0.108  0.495 | 0.291  0.062 | -0.045  0.779 | -0.153  0.333 | -0.182  0.248 | 0.102  0.522 | -0.212  0.179 | -0.121  0.446 | -0.057  0.721 | -0.164  0.299 | -0.058  0.717 | -0.180  0.255 | -0.240  0.126 |
|  | 26-49 | r  p | 0.206  0.124 | -0.169  0.210 | 0.137  0.311 | -0.025  0.852 | 0.188  0.161 | 0.153  0.255 | -0.108  0.424 | 0.120  0.373 | -0.021  0.876 | -0.097  0.473 | -0.036  0.790 | 0.068  0.614 | 0.149  0.269 | -0.103  0.445 | -0.049  0.715 |
|  | >= 50 | r  p | -0.154  0.317 | -0.315*  0.037 | -0.157  0.310 | -0.328*  0.030 | -0.388**  0.009 | -0.269  0.078 | 0.088  0.569 | -0.048  0.757 | -0.290  0.056 | 0.243  0.112 | 0.266  0.081 | -0.411**  0.006 | -0.326*  0.031 | 0.087  0.575 | -0.167  0.279 |
| Social isolation | Total | r  p | 0.093  0.272 | -0.060  0.475 | 0.139  0.098 | -0.093  0.270 | -0.077  0.358 | -0.100  0.236 | -0.073  0.383 | -0.035  0.676 | -0.057  0.495 | -0.074  0.379 | -0.032  0.704 | -0.044  0.598 | -0.105  0.211 | -0.075  0.372 | -0.066  0.432 |
|  | <= 25 | r  p | -0.072  0.651 | 0.074  0.642 | 0.346*  0.025 | -0.076  0.633 | -0.215  0.171 | -0.349*  0.023 | -0.083  0.600 | -0.175  0.269 | -0.259  0.098 | -0.181  0.252 | -0.207  0.188 | 0.463**  0.001 | -0.312*  0.044 | -0.092  0.562 | -0.280  0.073 |
|  | 26-49 | r  p | 0.139  0.302 | -0.152  0.259 | 0.105  0.436 | 0.005  0.970 | 0.068  0.613 | -0.110  0.416 | 0.074  0.586 | -0.068  0.614 | -0.094  0.487 | -0.130  0.334 | -0.094  0.487 | -0.002  0.990 | -0.049  0.717 | 0.071  0.599 | -0.112  0.409 |
|  | >= 50 | r  p | 0.242  0.114 | 0.097  0.531 | 0.039  0.802 | -0.058  0.710 | -0.001  0.996 | 0.035  0.824 | -0.122  0.429 | 0.147  0.342 | 0.134  0.386 | 0.077  0.619 | 0.116  0.453 | 0.095  0.540 | 0.022  0.890 | -0.117  0.449 | 0.145  0.349 |
| Social tensions | Total | r  p | 0.132  0.115 | -0.018  0.835 | 0.154  0.067 | -0.002  0.984 | 0.049  0.561 | 0.034  0.691 | 0.015  0.863 | 0.129  0.124 | -0.021  0.807 | -0.001  0.988 | 0.014  0.872 | -0.020  0.814 | 0.035  0.682 | 0.020  0.813 | -0.014  0.872 |
|  | <= 25 | r  p | 0.087  0.585 | -0.026  0.869 | 0.270  0.083 | -0.051  0.750 | -0.098  0.538 | -0.251  0.109 | -0.115  0.470 | 0.208  0.186 | 0.065  0.685 | -0.034  0.831 | 0.093  0.557 | 0.204  0.178 | -0.209  0.185 | -0.106  0.503 | 0.021  0.893 |
|  | 26-49 | r  p | 0.235  0.078 | -0.125  0.353 | 0.057  0.674 | 0.105  0.437 | 0.287*  0.030 | 0.371**  0.005 | 0.228  0.089 | 0.120  0.373 | -0.078  0.653 | -0.098  0.466 | -0.041  0.764 | -0.017  0.900 | 0.350**  0.008 | 0.235  0.079 | -0.083  0.540 |
|  | >= 50 | r  p | 0.171  0.268 | 0.138  0.372 | 0.276  0.069 | 0.013  0.933 | 0.023  0.880 | 0.067  0.666 | -0.128  0.409 | 0.078  0.613 | 0.029  0.852 | 0.168  0.276 | 0.165  0.284 | -0.022  0.888 | 0.061  0.692 | -0.126  0.417 | 0.079  0.611 |
| Screening Scale for chronic stress (SSCS) | Total | r  p | 0.143  0.092 | -0.014  0.869 | 0.211*  0.012 | -0.109  0.201 | -0.084  0.322 | -0.105  0.217 | -0.098  0.247 | 0.138  0.104 | -0.082  0.333 | -0.042  0.619 | -0.003  0.974 | -0.077  0.366 | -0.113  0.184 | -0.093  0.274 | -0.072  0.395 |
|  | <= 25 | r  p | 0.159  0.316 | 0.072  0.649 | 0.116  0.463 | 0.084  0.598 | -0.045  0.780 | -0.185  0.240 | -0.248  0.113 | 0.309*  0.046 | -0.063  0.691 | 0.035  0.824 | 0.090  0.569 | -0.072  0649 | -0.122  0.440 | -0.237  0.131 | -0.030  0.850 |
|  | 26-49 | r  p | 0.333*  0.013 | 0.015  0.914 | 0.379**  0.004 | -0.022  0.872 | 0.070  0.610 | -0.075  0.588 | 0.051  0.713 | 0.139  0.311 | -0.053  0.702 | -0.164  0.230 | -0.115  0.404 | 0.120  0.384 | -0.033  0.811 | 0.058  0.676 | -0.103  0.455 |
|  | >= 50 | r  p | 0.080  0.610 | 0.038  0.811 | -0.019  0.905 | -0.192  0.216 | -0.144  0.357 | -0.065  0.678 | -0.115  0.463 | 0.084  0.594 | -0.057  0.718 | 0.183  0.241 | 0.205  0.188 | -0.135  0.388 | -0.101  0.519 | -0.112  0.473 | 0.014  0.929 |

1: optical reaction time, 2: pursuing reaction time, 3: acoustical reaction time, 4: verbal reaction time, 5: cognitive reaction time, 6: cognitive switching capability, 7: ability to concentrate (time), 8: ability to concentrate (mistakes), 9: strategic thinking, 10: memory performance, 11: orientation capability, 12: changeover capability, **: the correlation is significant at the level 0.01 (2-sided), *: the correlation is significant at the level 0.05 (2-sided)
